# Hemoglobin Glycation Index and Prognosis in Patients With Acute Myocardial Infarction Without Reduced Left Ventricular Ejection Fraction: A Multicenter Retrospective Cohort Study

**DOI:** 10.1111/1753-0407.70257

**Published:** 2026-07-20

**Authors:** Shangjian Luo, Huan Liu, Xuesong Wen

**Affiliations:** ^1^ Department of Cardiovascular Medicine, Cardiovascular Research Center The First Affiliated Hospital of Chongqing Medical University Chongqing China; ^2^ Department of Ophthalmology, Chongqing Emergency Medical Center Chongqing University Central Hospital, School of Medicine, Chongqing University Chongqing China

**Keywords:** acute myocardial infarction, hemoglobin glycation index, left ventricular ejection fraction, prognosis, risk stratification

## Abstract

**Background:**

The prognostic value of the hemoglobin glycation index (HGI) in patients with acute myocardial infarction (AMI) without reduced left ventricular ejection fraction (LVEF) remains unclear.

**Methods:**

This multicenter retrospective cohort study included 1706 patients with AMI without reduced LVEF. HGI was calculated as measured HbA1c minus predicted HbA1c. Restricted cubic spline (RCS) analysis was used to evaluate nonlinear associations between HGI and outcomes and identify the cutoff value. Piecewise Cox regression and subgroup analyses were also performed. The primary endpoint was major adverse cardiovascular events (MACEs), defined as a composite of rehospitalization for heart failure (HF), recurrent myocardial infarction (MI), and cardiovascular death.

**Results:**

The median follow‐up was 3.86 (2.30–4.98) years. RCS analysis showed a non‐linear association between HGI and adverse outcomes, with a cutoff at −0.174. In patients with HGI ≤ −0.174, higher HGI was associated with lower risks of rehospitalization for HF (HR = 0.600, 95% CI: 0.464–0.776), recurrent MI (HR = 0.599, 95% CI: 0.365–0.983), cardiovascular death (HR = 0.627, 95% CI: 0.458–0.859), all‐cause death (HR = 0.552, 95% CI: 0.428–0.713), and MACEs (HR = 0.603, 95% CI: 0.483–0.753) after multivariable adjustment. In contrast, no significant associations were observed in patients with HGI > −0.174 after adjustment. Within the lower‐HGI subgroup, severely low HGI was associated with a higher risk of rehospitalization for HF and MACEs compared with mildly low HGI. Subgroup analyses were generally consistent with the main results.

**Conclusions:**

In AMI patients without reduced LVEF, HGI showed a non‐linear association with adverse outcomes, mainly in the lower‐HGI range. These exploratory findings require prospective validation before clinical application.

**Trial Registration:** ClinicalTrials.gov Identifier: NCT04485988 and NCT04564365

## Highlights

1. HGI showed a nonlinear association with adverse outcomes in AMI patients without reduced LVEF.
2. The prognostic effect of HGI was mainly concentrated in patients with lower HGI values.
3. Severely low HGI was associated with higher risks of heart failure rehospitalization and MACEs.

## Graphical abstract

Among patients with acute myocardial infarction without reduced left ventricular ejection fraction, hemoglobin glycation index showed a nonlinear association with prognosis. These exploratory findings require prospective validation before clinical application.

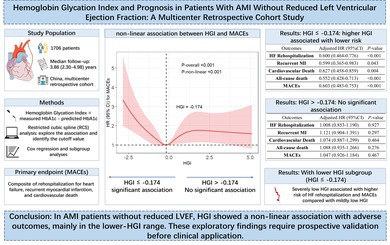

**Abbreviations**
ACEI: angiotensin‐converting enzyme inhibitor
ACS: acute coronary syndrome
AF: atrial fibrillation
AMI: acute myocardial infarction
ARB: angiotensin receptor blocker
ARNI: angiotensin receptor‐neprilysin inhibitor
BMI: body mass index
BNP: B‐type natriuretic peptide
CABG: coronary artery bypass grafting
CAD: coronary artery disease
CAG: coronary angiography
CI: confidence interval
CKD: chronic kidney disease
CK‐MB: creatine kinase‐MB
cTnI: cardiac troponin I
DAPT: dual antiplatelet therapy
DBP: diastolic blood pressure
eGFR: estimated glomerular filtration rate
FBG: fasting blood glucose
HbA1c: glycated hemoglobin
HF: heart failure
HGI: hemoglobin glycation index
HR: hazard ratio
IQR: interquartile range
LDL‐C: low‐density lipoprotein cholesterol
LVEF: left ventricular ejection fraction
MACEs: major adverse cardiovascular events
MI: myocardial infarction
PCI: percutaneous coronary intervention
RCS: restricted cubic spline
SBP: systolic blood pressure
SHR: stress hyperglycemia ratio
T2DM: type 2 diabetes mellitus

## 1 Introduction

Acute myocardial infarction (AMI) remains a major cause of adverse cardiovascular outcomes despite advances in reperfusion therapy and secondary prevention [1]. Left ventricular ejection fraction (LVEF) is widely used for risk stratification after AMI, and patients without reduced LVEF are often considered to have a relatively better prognosis [1]. However, these patients are not truly low risk. Adverse events after discharge, including heart failure (HF), recurrent myocardial infarction (MI), and death, are still common in this population [2]. Better markers are needed to identify those who remain at increased risk after discharge.

Abnormal glucose metabolism is common in AMI and has been associated with poor prognosis [3]. Fasting blood glucose (FBG) and glycated hemoglobin (HbA1c) are the two markers most commonly used in clinical practice, but each reflects a different aspect of glycemic status. FBG may be influenced by acute stress during AMI, whereas HbA1c mainly reflects chronic glycemic exposure over the previous 2–3 months [3, 4]. The hemoglobin glycation index (HGI), defined as the difference between measured HbA1c and predicted HbA1c, was proposed to reflect the individual discordance between blood glucose and hemoglobin glycation [5]. It may therefore provide information beyond FBG or HbA1c alone.

Previous studies have examined the association between HGI and adverse outcomes in diabetes and cardiovascular disease [6, 7]. Some have suggested that the relationship may be non‐linear, with increased risk at both low and high HGI levels [8]. Evidence in patients with AMI without reduced LVEF remains limited. Most studies have focused on diabetes, acute coronary syndrome (ACS), or critically ill AMI populations rather than this specific subgroup [8, 9, 10]. Whether HGI shows a similar non‐linear pattern in AMI patients without reduced LVEF is still uncertain. It is also unclear whether the prognostic signal is distributed across the whole range of HGI or mainly concentrated in a specific interval.

In this multicenter retrospective cohort study, we examined the association between HGI and outcomes in AMI patients without reduced LVEF. We also explored the potential nonlinear relationship between HGI and adverse events using restricted cubic spline (RCS) analysis, and further assessed the prognostic significance of HGI across different ranges.

## 2 Methods

### 2.1 Study Design

This was a multicenter retrospective cohort study based on data from two registered clinical cohorts (ClinicalTrials.gov identifiers: NCT04485988 and NCT04564365). Consecutive patients admitted between October 2012 and April 2021 were screened. Epidemiological data, comorbidities, angiographic characteristics, laboratory results, and prescribed medication information were collected from the medical records. All patients completed clinical follow‐up through face‐to‐face interviews, telephone follow‐ups, or medical record reviews. The study was approved by the local ethics committee (approval number: 2020‐607) and conducted in accordance with the Declaration of Helsinki.

### 2.2 Study Population

**FIGURE 1:**
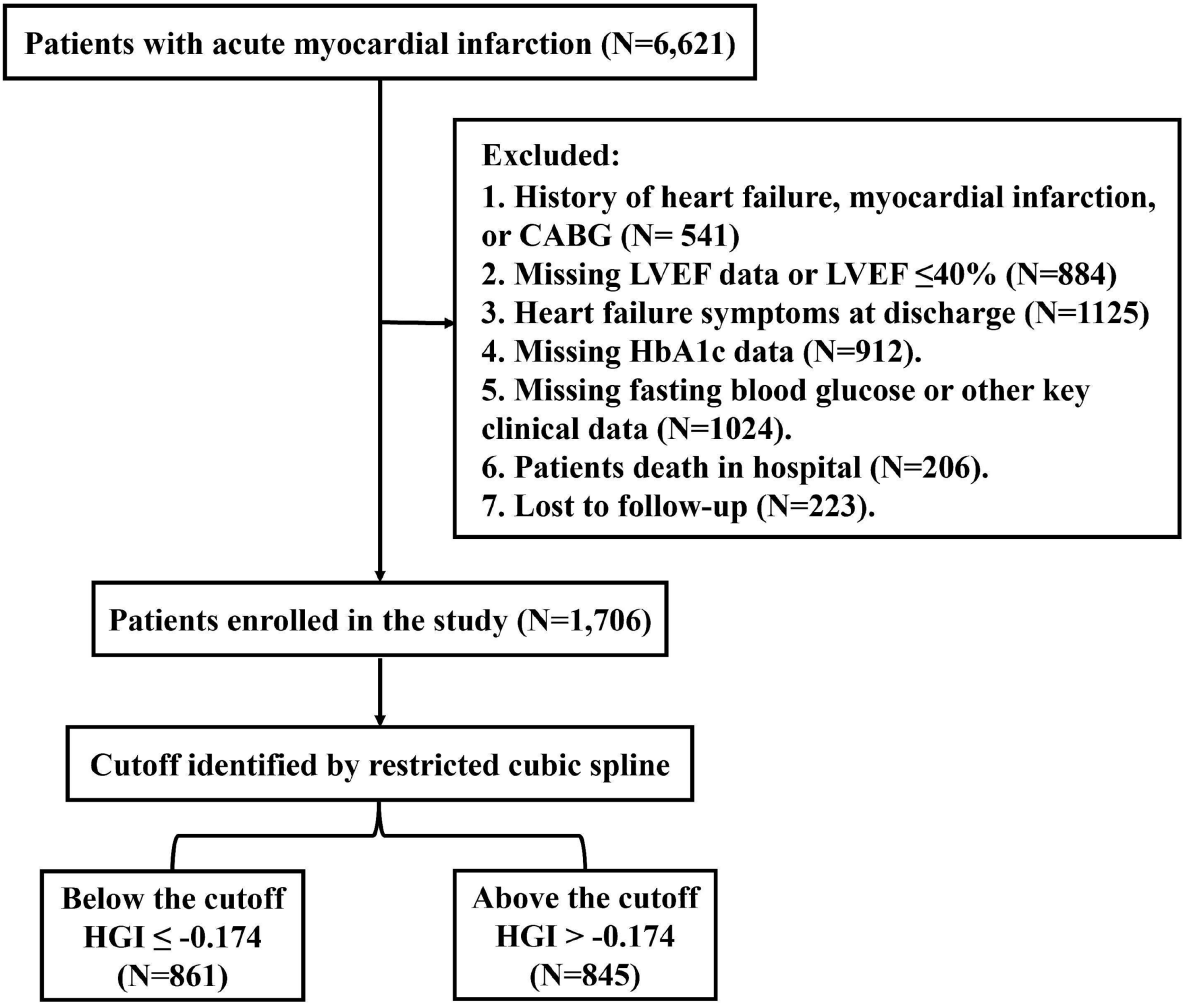
Flowchart of patient selection and HGI grouping. Abbreviations: HbA1c, glycated hemoglobin; HGI, hemoglobin glycation index; LVEF, left ventricular ejection fraction.

Patients with AMI without reduced LVEF were eligible for inclusion. AMI was diagnosed and classified according to the criteria of the Fourth Universal Definition of Myocardial Infarction (2018) [11]. Reduced LVEF was defined as LVEF ≤ 40%, consistent with current guideline definitions of heart failure with reduced ejection fraction. Accordingly, patients without reduced LVEF (LVEF > 40%) comprised two categories: preserved ejection fraction (LVEF ≥ 50%) and mildly reduced ejection fraction (LVEF 41%–49%). In the study, a total of 6621 AMI patients were screened. Exclusion criteria were as follows: prior history of heart failure (HF), MI, or coronary artery bypass grafting (CABG); missing LVEF data or LVEF ≤ 40%; signs or symptoms of HF at discharge; missing HbA1c data; missing FBG or other key clinical data; in‐hospital death; and no available follow‐up data. Ultimately, 1706 patients were included in the final analysis. The study flowchart is shown in Figure 1.

### 2.3 Data Collection

Baseline data included demographic and clinical characteristics, comorbidities, angiographic characteristics, laboratory measurements, and medications at discharge. Specifically, the collected variables included age, sex, body mass index (BMI), heart rate (HR), systolic blood pressure (SBP), diastolic blood pressure (DBP), hypertension, type 2 diabetes mellitus (T2DM), chronic kidney disease (CKD), hyperlipidemia, coronary artery disease (CAD), stroke, smoking, cancer, atrial fibrillation (AF), STEMI, infarct location, Killip class, coronary angiography, thrombolytic therapy, percutaneous coronary intervention (PCI), primary PCI, CABG, reperfusion, LVEF, peak CK‐MB, peak cardiac troponin I, B‐type natriuretic peptide, FBG, HbA1c, HGI, creatinine, estimated glomerular filtration rate (eGFR, calculated using the CKD‐EPI equation), low‐density lipoprotein cholesterol (LDL‐c), and discharge medications. HGI was calculated subsequently as described below.

### 2.4 Outcomes

The primary endpoint was major adverse cardiovascular events (MACEs), defined as a composite of rehospitalization for HF, recurrent MI, and cardiovascular death. Secondary endpoints included rehospitalization for HF, recurrent MI, cardiovascular death, and all‐cause death.

### 2.5 Calculation of HGI

HGI was calculated as the difference between the measured HbA1c and the predicted HbA1c. Predicted HbA1c was derived from a simple linear regression model in which the measured HbA1c (%) was regressed on the corresponding fasting blood glucose (FBG, mmol/L) across the entire study population, as described previously [7]. The fitted regression equation (*R* ^2^ = 0.47, *p* < 0.001; Figure S1) was:

$$\text{Predicted}\;\mathrm{HbA}1c\;\left(\%\right)=4.312+0.304\times \mathrm{FBG}\left(\frac{\text{mmol}}{L}\right)$$

Accordingly, HGI (%) was calculated as follows:

$$\mathrm{HGI}\;\left(\%\right)=\text{measured}\;\mathrm{HbA}1c\;\left(\%\right)-\text{predicted}\;\mathrm{HbA}1c\;\left(\%\right)$$

A positive HGI indicates a measured HbA1c higher than that predicted from FBG, whereas a negative HGI indicates a measured HbA1c lower than predicted.

### 2.6 Statistical Analysis

Categorical variables are presented as counts and percentages and were compared using the chi‐square test or Fisher's exact test. Continuous variables are presented as mean ± standard deviation or median (*Q*1–*Q*3) and were compared using the Student's *t*‐test or Mann–Whitney U test. Simple linear regression was used to model the relationship between FBG and HbA1c and to derive predicted HbA1c for HGI calculation. The potential non‐linear relationship between HGI and outcomes was explored using restricted cubic spline (RCS) analysis, adjusting for age, sex, BMI, hypertension, T2DM, hyperlipidemia, CKD, STEMI, LVEF, and primary PCI. The number of knots was determined based on the minimum akaike information criterion. For subsequent stratified analyses, the HGI cutoff was defined as the value at the inflection point identified in the multivariable adjusted RCS curves for the study endpoints. Patients were then classified into lower‐ and higher‐HGI groups using this threshold. The association between HGI and outcomes was assessed using Cox proportional hazards models. Four models were constructed: Model 1 was unadjusted; Model 2 was adjusted for age, sex, and BMI; Model 3 was further adjusted for hypertension, T2DM, hyperlipidemia, CKD, STEMI, LVEF, and primary PCI; Model 4 was an extension of Model 3, with additional adjustment for peak cardiac troponin I, Killip class ≥ II, ACEI/ARB/ARNI, beta‐blocker, serum creatinine, BNP, thrombolytic therapy, and reperfusion therapy. Subgroup analyses and interaction tests were performed according to prespecified variables. Hazard ratios and 95% confidence intervals were calculated. All analyses were performed using R version 4.4.1 (R Foundation for Statistical Computing, Vienna, Austria). A two‐sided *p* value < 0.05 was considered statistically significant.

## 3 Results

### 3.1 Baseline Characteristics of the Study Population

A total of 1706 patients with AMI without reduced LVEF were included in the final analysis. Across the multivariable adjusted RCS curves for all study endpoints, a consistent inflection point was observed around HGI = −0.174. This value was therefore used as the cutoff for subsequent analyses, classifying 861 patients into the lower‐HGI group (HGI ≤ −0.174) and 845 into the higher‐HGI group (HGI > −0.174). The median follow‐up was 3.86 (2.30–4.98) years. Among the 1706 patients, 1434 (84.1%) had preserved LVEF (≥ 50%) and 272 (15.9%) had mildly reduced LVEF (41%–49%). Baseline characteristics are summarized in Table 1. Compared with the higher‐HGI group, patients in the lower‐HGI group had a lower prevalence of T2DM and hypertension, were more likely to present with STEMI and undergo primary PCI, and had lower HbA1c levels. In addition, they showed higher peak CK‐MB and lower BNP levels. Many other baseline characteristics were comparable between the two groups. At discharge, patients in the lower‐HGI group were less likely to receive ACEI/ARB/ARNI therapy or beta‐blockers.

**TABLE 1:** Baseline characteristics of the study population according to HGI groups.

Variables	Overall *N* = 1706	Lower HGI *N* = 861	Higher HGI *N* = 845	*p*
Demographic and clinical characteristics				
Age (years)	63.2 ± 12.3	63.5 ± 12.6	62.9 ± 12.0	0.325
Male, *n* (%)	1300 (76.2%)	658 (76.4%)	642 (76.0%)	0.873
BMI (kg/m^2^)	24.38 ± 3.14	24.05 ± 3.10	24.71 ± 3.15	< 0.001
HR (bpm)	80.8 ± 16.4	80.8 ± 16.5	80.9 ± 16.3	0.897
SBP (mmHg)	130.9 ± 24.9	130.8 ± 26.0	131.0 ± 23.8	0.901
DBP (mmHg)	78.0 (69.0–88.0)	78.0 (69.0–88.0)	78.0 (68.0–88.0)	0.292
Comorbidities				
Hypertension, *n* (%)	986 (57.8%)	471 (54.7%)	515 (60.9%)	0.010
T2DM, *n* (%)	717 (42.0%)	217 (25.2%)	500 (59.2%)	< 0.001
CKD, *n* (%)	122 (7.2%)	64 (7.4%)	58 (6.9%)	0.717
Hyperlipidemia, *n* (%)	338 (19.8%)	176 (20.4%)	162 (19.2%)	0.511
CAD, *n* (%)	66 (3.9%)	33 (3.8%)	33 (3.9%)	1.000
Stroke, *n* (%)	135 (7.9%)	58 (6.7%)	77 (9.1%)	0.084
Smoking, *n* (%)	1053 (61.7%)	532 (61.8%)	521 (61.7%)	0.995
Cancer, *n* (%)	38 (2.2%)	19 (2.2%)	19 (2.2%)	1.000
AF, *n* (%)	94 (5.5%)	46 (5.3%)	48 (5.7%)	0.842
Angiographic characteristics				
STEMI, *n* (%)	1196 (70.1%)	631 (73.3%)	565 (66.9%)	0.004
Anterior MI, *n* (%)	683 (40.0%)	349 (40.5%)	334 (39.5%)	0.707
Inf/Post MI, *n* (%)	592 (34.7%)	315 (36.6%)	277 (32.8%)	0.110
Other sites MI, *n* (%)	199 (11.7%)	115 (13.4%)	84 (9.9%)	0.034
Killip class ≥ II, *n* (%)	313 (18.3%)	162 (18.8%)	151 (17.9%)	0.659
CAG, *n* (%)	1580 (92.6%)	795 (92.3%)	785 (92.9%)	0.724
Thrombolytic, *n* (%)	60 (3.5%)	27 (3.1%)	33 (3.9%)	0.465
PCI therapy, *n* (%)	1371 (80.4%)	691 (80.3%)	680 (80.5%)	0.958
Primary PCI, *n* (%)	848 (49.7%)	457 (53.1%)	391 (46.3%)	0.006
CABG, *n* (%)	6 (0.4%)	2 (0.2%)	4 (0.5%)	0.448
Reperfusion, *n* (%)	1383 (81.1%)	698 (81.1%)	685 (81.1%)	1.000
LVEF (%)	56.7 ± 7.1	56.7 ± 7.1	56.6 ± 7.1	0.878
LVEF ≥ 50%, *n* (%)	1434.0 (84.1%)	726.0 (84.3%)	708.0 (83.8%)	0.763
Laboratory measurements				
Peak CK‐MB (μg/L)	15.6 (3.6–52.0)	19.4 (4.3–64.3)	12.1 (3.1–40.5)	< 0.001
Peak cTnI (ng/mL)	1.5 (0.2–8.2)	1.8 (0.2–9.1)	1.2 (0.3–7.4)	0.204
BNP (pg/mL)	177.0 (79.1–397.5)	163.0 (71.2–337.0)	198.0 (86.0–464.0)	0.001
FBG (mmol/L)	6.9 (5.7–9.3)	7.0 (5.8–9.0)	6.7 (5.6–9.7)	0.051
HbA1c (%)	6.2 (5.7–7.3)	5.8 (5.5–6.2)	6.9 (6.2–8.5)	< 0.001
HGI (%)	−0.2 (−0.6–0.3)	−0.6 (−1.0–0.4)	0.3 (0.0–1.1)	< 0.001
Creatinine (μmol/L)	75.0 (64.0–91.0)	75.0 (64.0–92.0)	75.0 (63.0–91.0)	0.492
eGFR (mL/min/1.73 m^2^)	93.0 (74.1–103.3)	92.6 (73.2–103.0)	93.5 (74.7–103.4)	0.659
LDL‐C (mmol/L)	2.80 ± 0.93	2.82 ± 0.94	2.78 ± 0.92	0.396
Medications at discharge				
Anticoagulants, *n* (%)	41 (2.4%)	18 (2.1%)	23 (2.7%)	0.488
Diuretic, *n* (%)	303 (17.8%)	152 (17.7%)	151 (17.9%)	0.957
Antiplatelets, *n* (%)	1572 (92.1%)	784 (91.1%)	788 (93.3%)	0.110
Clopidogrel/Ticagrelor	1676 (98.2%)	846 (98.3%)	830 (98.2%)	1.000
DAPT, *n* (%)	1550 (90.9%)	771 (89.5%)	779 (92.2%)	0.070
Statin, *n* (%)	1678 (98.4%)	848 (98.5%)	830 (98.2%)	0.810
ACEI/ARB/ARNI	1209 (70.9%)	589 (68.4%)	620 (73.4%)	0.028
Beta‐blocker, *n* (%)	1411 (82.7%)	695 (80.7%)	716 (84.7%)	0.033

*Note:* Data are presented as mean ± standard deviation (SD), median (interquartile range [IQR]), or number (percentage). Comparisons between groups were performed using the Student's *t*‐test or Wilcoxon rank‐sum test for continuous variables, and the chi‐squared test or Fisher's exact test for categorical variables. Lower HGI: HGI ≤ −0.174; Higher HGI: HGI > −0.174.

Abbreviations: ACEI, angiotensin‐converting enzyme inhibitor; AF, atrial fibrillation; ARB, angiotensin receptor blocker; ARNI, angiotensin receptor‐neprilysin inhibitor; BMI, body mass index; BNP, B‐type natriuretic peptide; CABG, coronary artery bypass grafting; CAD, coronary artery disease; CAG, coronary angiography; CKD, chronic kidney disease; CK‐MB, creatine kinase‐MB; cTnI, cardiac troponin I; DAPT, dual antiplatelet therapy; eGFR, estimated glomerular filtration rate; Glu, glucose; HbA1c, glycated hemoglobin; HGI, hemoglobin glycation index; LDL‐C, low‐density lipoprotein cholesterol; LVEF, left ventricular ejection fraction; PCI, percutaneous coronary intervention; T2DM, type 2 diabetes mellitus.

In addition, to assess potential selection bias, we compared the baseline characteristics of the excluded patients with those of the included cohort (Table S1). Most variables were comparable between the two groups, although differences were observed in BMI, diabetes status, STEMI presentation, reperfusion therapy, BNP, and discharge medications. Given the retrospective design and the presence of some baseline differences, the possibility of residual selection bias cannot be completely excluded. We have acknowledged this issue as a limitation.

### 3.2 Nonlinear Associations Between HGI and Outcomes

**FIGURE 2:**
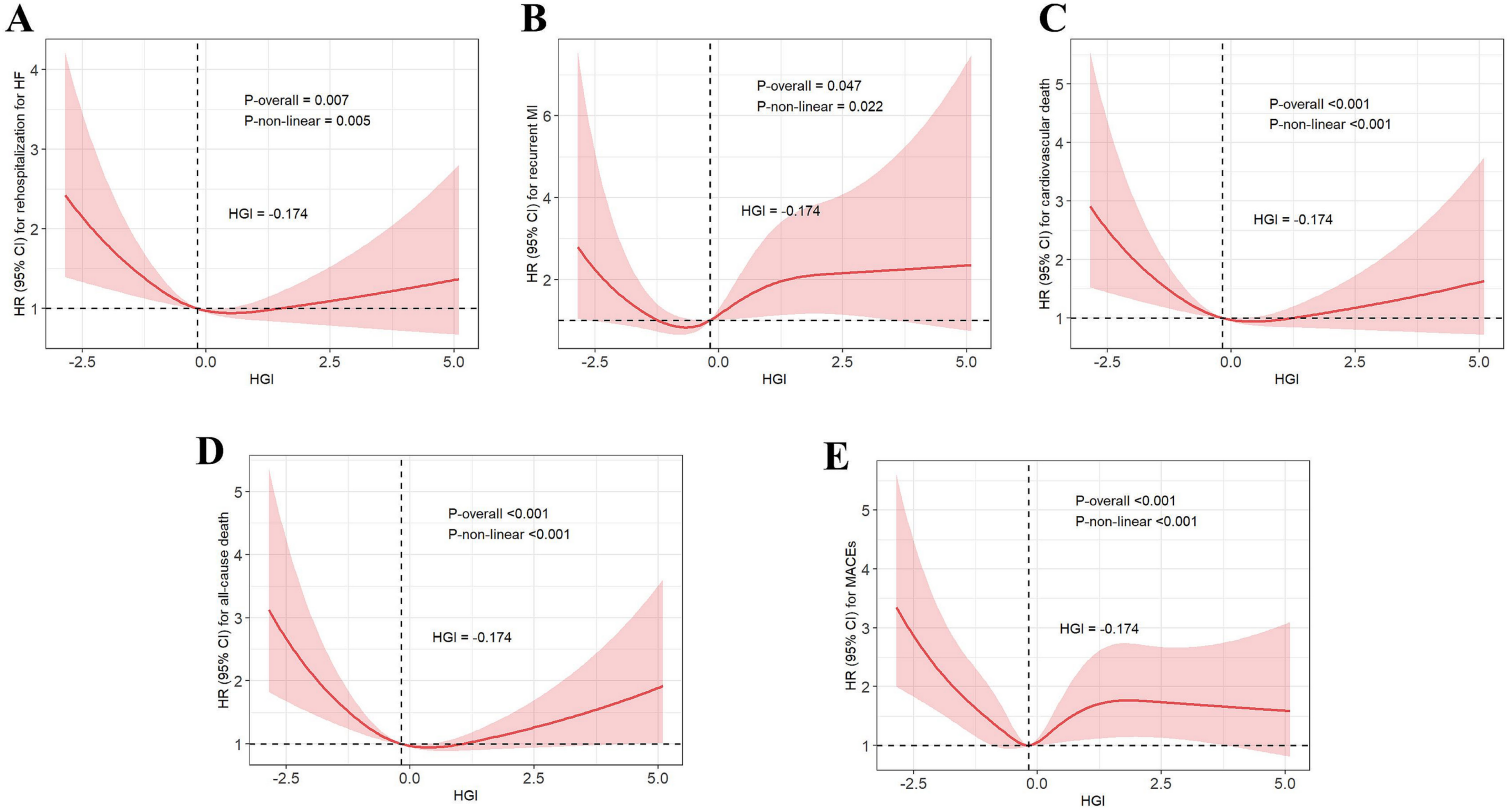
Adjusted restricted cubic spline curves for the associations of HGI with outcomes. RCS analyses of the associations between HGI and rehospitalization for heart failure (A), recurrent myocardial infarction (B), cardiovascular death (C), all‐cause death (D), and MACEs (E). The model was adjusted for age, sex, BMI, hypertension, T2DM, hyperlipidemia, CKD, STEMI, LVEF, and primary PCI. Abbreviations: CI, confidence interval; HGI, hemoglobin glycation index; HR, hazard ratio; MACEs, major adverse cardiovascular events.

RCS analyses demonstrated significant nonlinear associations between HGI and outcomes (Figure 2). The overall and nonlinear associations were both significant for rehospitalization for HF (*p*‐overall = 0.007, *p*‐nonlinear = 0.005), recurrent MI (*p*‐overall = 0.047, *p*‐nonlinear = 0.022), cardiovascular death (both *p* < 0.001), all‐cause death (both *p* < 0.001), and MACEs (both *p* < 0.001). Across all endpoints, the spline curves generally showed a similar pattern, with an apparent inflection around an HGI value of −0.174. Below this value, the hazard progressively increased as HGI decreased, whereas the risk gradient above the cutoff appeared less pronounced. On the basis of these findings, an HGI value of −0.174 was used as the cutoff for subsequent analyses.

### 3.3 Associations of HGI With Outcomes Below and Above the Cutoff

Piecewise Cox regression analyses were performed to further evaluate the associations between HGI and outcomes on either side of the cutoff (Table 2). In patients with HGI ≤ −0.174, lower HGI levels were consistently associated with a higher risk of adverse outcomes. In the adjusted model (Model 3), each 1‐unit increase in HGI was associated with lower hazards of rehospitalization for HF (HR = 0.600, 95% CI: 0.464–0.776), recurrent MI (HR = 0.599, 95% CI: 0.365–0.983), cardiovascular death (HR = 0.627, 95% CI: 0.458–0.859), all‐cause death (HR = 0.552, 95% CI: 0.428–0.713), and MACEs (HR = 0.603, 95% CI: 0.483–0.753). Similar trends were observed in the unadjusted (Model 1) and partially adjusted model (Model 2).

**TABLE 2:** Associations of HGI with outcomes below and above the cutoff point: Piecewise Cox regression analyses.

Variables	Lower HGI (HGI ≤ −0.174)		Higher HGI (HGI > −0.174)	
	HR (95% CI)	*p*	HR (95% CI)	*p*
Rehospitalization for HF				
Model 1	0.637 (0.507–0.801)	< 0.001	1.092 (0.956–1.248)	0.196
Model 2	0.603 (0.478–0.759)	< 0.001	1.096 (0.957–1.256)	0.185
Model 3	0.600 (0.464–0.776)	< 0.001	1.008 (0.853–1.190)	0.927
Model 4	0.601 (0.457–0.790)	< 0.001	1.014 (0.852–1.208)	0.873
Recurrent MI				
Model 1	0.648 (0.415–1.010)	0.055	1.126 (0.939–1.350)	0.201
Model 2	0.667 (0.420–1.059)	0.086	1.151 (0.955–1.386)	0.140
Model 3	0.599 (0.365–0.983)	0.043	1.121 (0.904–1.391)	0.297
Model 4	0.645 (0.378–1.100)	0.108	1.115 (0.902–1.378)	0.315
Cardiovascular death				
Model 1	0.652 (0.495–0.860)	0.002	1.103 (0.941–1.293)	0.226
Model 2	0.655 (0.493–0.871)	0.004	1.102 (0.938–1.295)	0.237
Model 3	0.627 (0.458–0.859)	0.004	1.074 (0.887–1.299)	0.464
Model 4	0.687 (0.489–0.965)	0.031	1.118 (0.928–1.348)	0.241
All‐cause death				
Model 1	0.602 (0.480–0.754)	< 0.001	1.106 (0.969–1.262)	0.137
Model 2	0.602 (0.477–0.760)	< 0.001	1.098 (0.962–1.253)	0.168
Model 3	0.552 (0.428–0.713)	< 0.001	1.088 (0.935–1.266)	0.276
Model 4	0.599 (0.454–0.791)	< 0.001	1.115 (0.961–1.294)	0.152
Major adverse cardiovascular events				
Model 1	0.636 (0.520–0.777)	< 0.001	1.108 (1.002–1.225)	0.046
Model 2	0.614 (0.499–0.755)	< 0.001	1.113 (1.005–1.232)	0.040
Model 3	0.603 (0.483–0.753)	< 0.001	1.047 (0.926–1.184)	0.467
Model 4	0.633 (0.502–0.798)	< 0.001	1.058 (0.933–1.199)	0.382

*Note:* Model 1 was unadjusted; Model 2 was adjusted for age, sex, and BMI; Model 3 was further adjusted for hypertension, T2DM, hyperlipidemia, CKD, STEMI, LVEF, and primary PCI; and Model 4 was further adjusted for cTnI, Killip class≥ II, ACEI/ARB/ARNI, β‐blocker, creatinine, BNP, thrombolytic therapy, reperfusion therapy.

Abbreviations: CI, confidence interval; HF, heart failure; HGI, hemoglobin glycation index; HR, hazard ratio; MI, myocardial infarction.

In contrast, among patients with HGI > −0.174, the associations were attenuated after multivariable adjustment. In the fully adjusted model, HGI was not significantly associated with rehospitalization for HF (HR = 1.008, 95% CI: 0.853–1.190), recurrent MI (HR = 1.121, 95% CI: 0.904–1.391), cardiovascular death (HR = 1.074, 95% CI: 0.887–1.299), all‐cause death (HR = 1.088, 95% CI: 0.935–1.266), or MACEs (HR = 1.047, 95% CI: 0.926–1.184). Although a modest association with MACEs was observed in the less adjusted models, it was no longer evident after full adjustment.

To examine whether the associations observed in the lower‐HGI range were robust to further covariate adjustment, we additionally extended Models 3 to Model 4. In patients with HGI ≤ −0.174, each 1‐unit increase in HGI remained associated with a lower risk of rehospitalization for HF (HR = 0.601, 95% CI: 0.457–0.790), cardiovascular death (HR = 0.687, 95% CI: 0.489–0.965), all‐cause death (HR = 0.599, 95% CI: 0.454–0.791), and MACEs (HR = 0.633, 95% CI: 0.502–0.798). The association with recurrent MI was attenuated and was no longer statistically significant (HR = 0.645, 95% CI: 0.378–1.100). Among patients with HGI > −0.174, no significant associations were observed after this additional adjustment.

### 3.4 Kaplan–Meier and Cox Regression Analyses in Patients With Lower HGI

**FIGURE 3:**
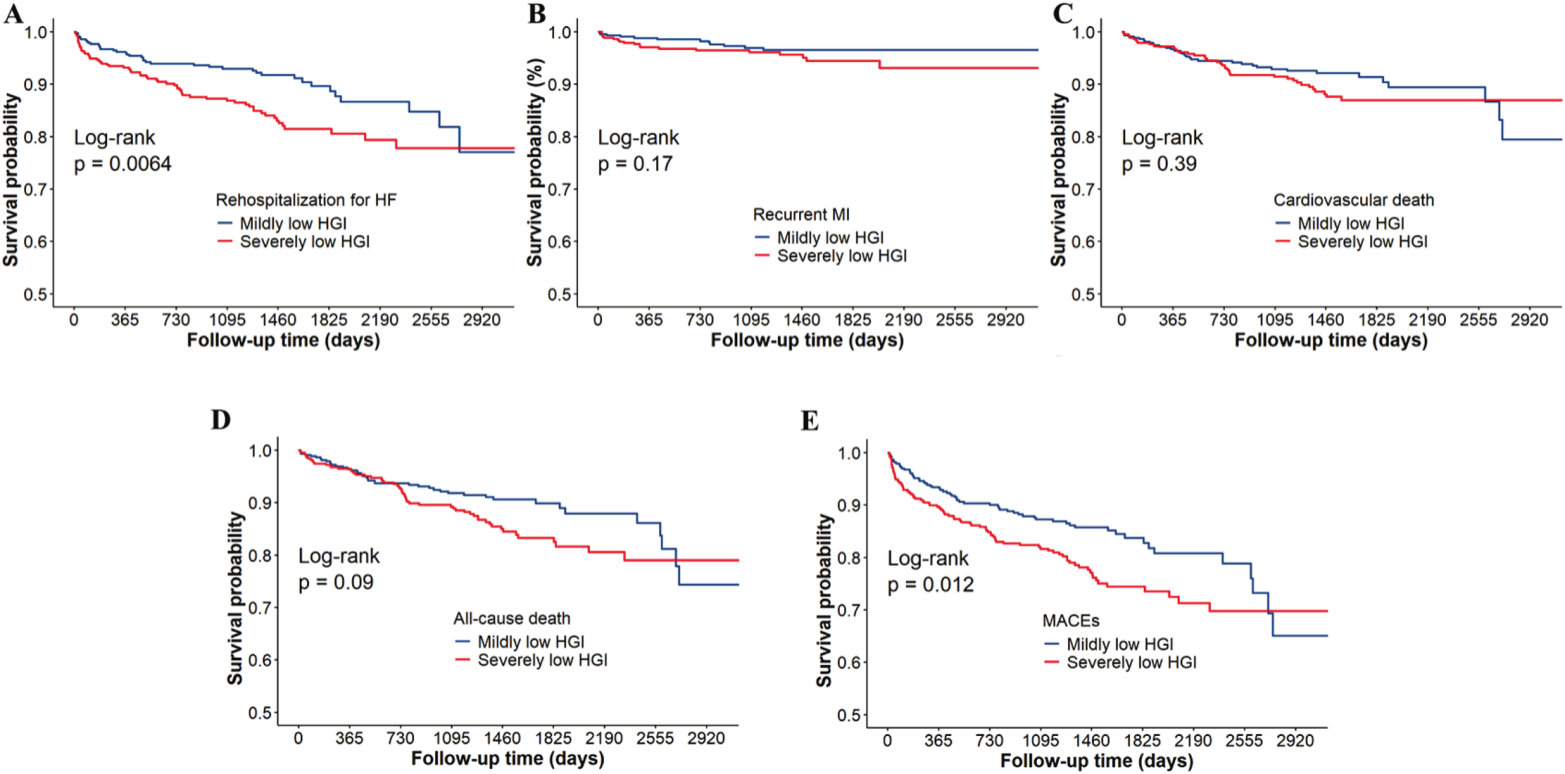
Kaplan–Meier curves for outcomes in patients with HGI ≤ −0.174. Kaplan–Meier curves are shown for rehospitalization for HF (A), recurrent MI (B), cardiovascular death (C), all‐cause death (D), and MACEs (E). Patients were divided into mildly low HGI (−0.623 < HGI ≤ −0.174) and severely low HGI (HGI ≤ −0.623) groups according to the median HGI within the lower‐HGI subgroup. Abbreviations: HGI, hemoglobin glycation index; MACEs, major adverse cardiovascular events.

Further analyses were therefore focused on patients with HGI ≤ −0.174, in whom the prognostic association was most evident. This subgroup was divided into mildly low HGI (−0.623 < HGI ≤ −0.174, *n* = 428) and severely low HGI (HGI ≤ −0.623, *n* = 433) according to the median HGI value within the lower‐HGI range. Kaplan–Meier analyses showed that patients with severely low HGI had a significantly higher cumulative incidence of rehospitalization for HF (log‐rank *p* = 0.006) and MACEs (log‐rank *p* = 0.012) than those with mildly low HGI (Figure 3). By contrast, the differences in recurrent MI (log‐rank *p* = 0.17), cardiovascular death (log‐rank *p* = 0.39), and all‐cause death (log‐rank *p* = 0.09) did not reach statistical significance.

The results of Cox regression analysis were consistent with the Kaplan–Meier findings (Table 3). In the fully adjusted model, severely low HGI was associated with a higher risk of rehospitalization for HF (HR = 1.682, 95% CI: 1.117–2.534) and MACEs (HR = 1.490, 95% CI: 1.071–2.074) compared with mildly low HGI. A numerical increase was also observed for all‐cause death (HR = 1.460, 95% CI: 0.966–2.205), although this did not reach statistical significance. No significant associations were found for recurrent MI (HR = 1.685, 95% CI: 0.800–3.549) or cardiovascular death (HR = 1.213, 95% CI: 0.759–1.940).

**TABLE 3:** Cox regression analysis of lower‐tail HGI subgroups and outcomes among patients with HGI ≤ −0.174.

Variables	Mildly low HGI (−0.623 < HGI ≤ −0.174) (*N* = 428)	Severely low HGI (HGI ≤ −0.623) (*N* = 433)	
	HR (95% CI)	HR (95% CI)	*p*
Rehospitalization for HF			
Model 1	Ref.	1.728 (1.160–2.574)	0.007
Model 2	Ref.	1.795 (1.201–2.682)	0.004
Model 3	Ref.	1.682 (1.117–2.534)	0.013
Recurrent MI			
Model 1	Ref.	1.652 (0.802–3.403)	0.174
Model 2	Ref.	1.588 (0.765–3.294)	0.215
Model 3	Ref.	1.685 (0.800–3.549)	0.170
Cardiovascular death			
Model 1	Ref.	1.217 (0.775–1.911)	0.394
Model 2	Ref.	1.212 (0.769–1.911)	0.408
Model 3	Ref.	1.213 (0.759–1.940)	0.420
All‐cause death			
Model 1	Ref.	1.409 (0.946–2.100)	0.092
Model 2	Ref.	1.404 (0.939–2.099)	0.099
Model 3	Ref.	1.460 (0.966–2.205)	0.073
Major adverse cardiovascular events			
Model 1	Ref.	1.503 (1.091–2.071)	0.013
Model 2	Ref.	1.530 (1.107–2.114)	0.010
Model 3	Ref.	1.490 (1.071–2.074)	0.018

*Note:* The mildly low HGI group was used as the reference group. Model 1 was unadjusted; Model 2 was adjusted for age, sex, and BMI; and Model 3 was further adjusted for hypertension, T2DM, hyperlipidemia, CKD, STEMI, LVEF, and primary PCI.

Abbreviations: CI, confidence interval; HF, heart failure; HGI, hemoglobin glycation index; HR, hazard ratio; MI, myocardial infarction.

### 3.5 Subgroup Analyses in Patients With Lower HGI

**FIGURE 4:**
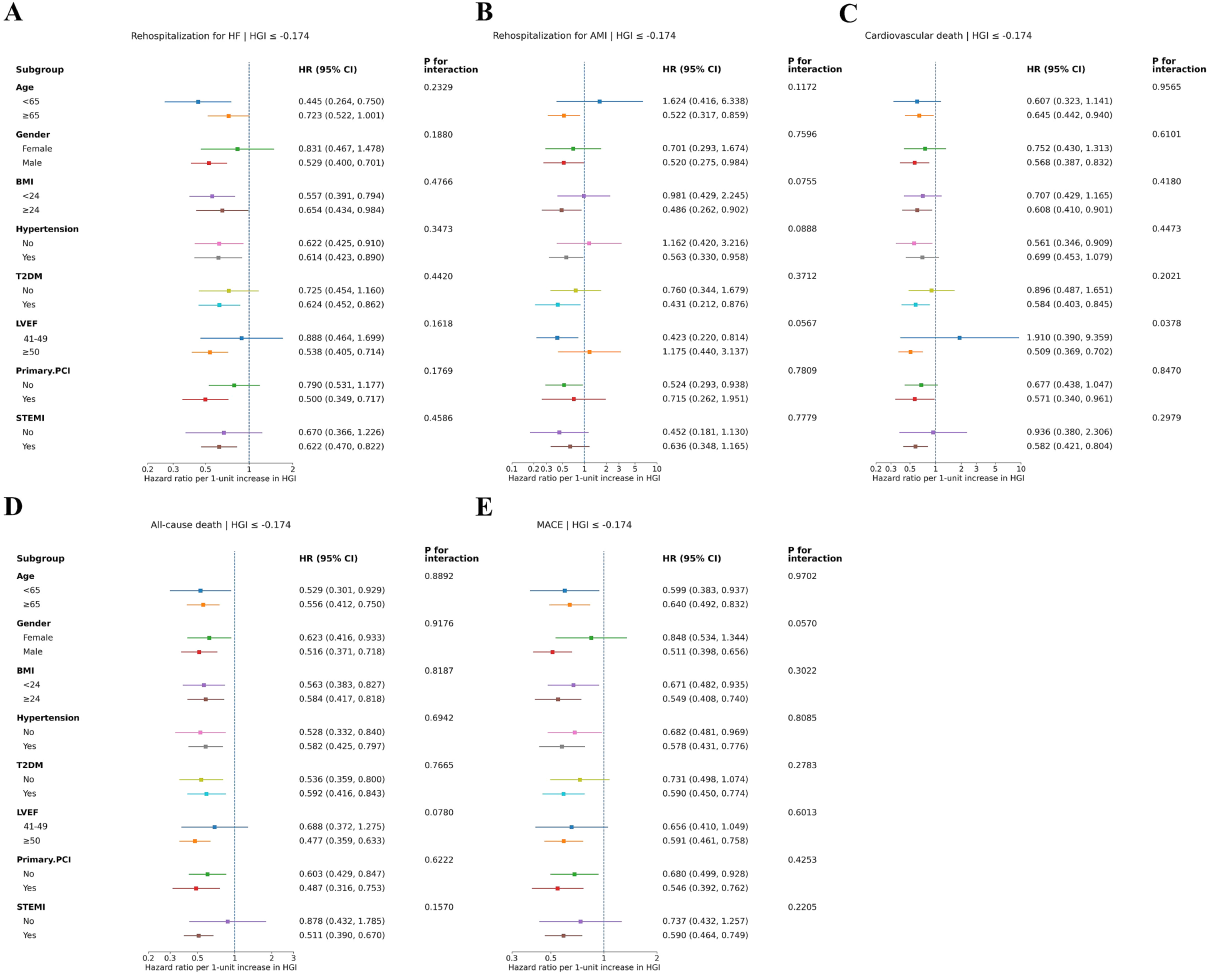
Subgroup analyses of outcomes in patients with HGI ≤ −0.174. Forest plots of subgroup analyses for rehospitalization for heart failure (A), recurrent myocardial infarction (B), cardiovascular death (C), all‐cause death (D), and MACEs (E) among patients with HGI ≤ −0.174. Hazard ratios with 95% confidence intervals are presented per 1‐unit increase in HGI. *P* for interaction is shown for each subgroup. The model was adjusted for age, sex, BMI, hypertension, T2DM, hyperlipidemia, CKD, STEMI, LVEF, and primary PCI. Abbreviations: CI, confidence interval; HGI, hemoglobin glycation index; HR, hazard ratio; MACEs, major adverse cardiovascular events.

Subgroup analyses were performed to assess whether the associations between HGI and outcomes within the lower‐HGI range were consistent across subgroups (Figure 4). Overall, the inverse associations between HGI and adverse outcomes were broadly consistent across age, sex, BMI, hypertension, T2DM, primary PCI, and STEMI subgroups. No significant interaction was observed for rehospitalization for HF, recurrent MI, all‐cause death, or MACEs.

For cardiovascular death, there was a significant interaction with LVEF category (*p* for interaction = 0.038). In patients with LVEF ≥ 50%, higher HGI within the lower‐HGI range was associated with a lower risk of cardiovascular death (HR = 0.509, 95% CI: 0.369–0.702). This association was not observed in those with LVEF 41%–49% (HR = 1.910, 95% CI: 0.390–9.359). No significant interaction was found for the other subgroup analyses.

### 3.6 Supplementary Analyses in Patients With Higher HGI

Additional analyses were also performed in patients with HGI > −0.174. This subgroup was further divided into mildly high HGI (−0.174 < HGI < 0.326, *n* = 423) and severely high HGI (HGI ≥ 0.326, *n* = 422). Kaplan–Meier curves showed a significant difference only for MACEs (log‐rank *p* = 0.016), while the differences between the groups were not significant for rehospitalization for HF (log‐rank *p* = 0.071), recurrent MI (log‐rank *p* = 0.21), cardiovascular death (log‐rank *p* = 0.30), or all‐cause death (log‐rank *p* = 0.37) (Figure S2).

Consistent with these findings, Cox regression analyses did not show an independent association between severely high HGI and adverse outcomes after multivariable adjustment (Table S2). In the fully adjusted model, the HRs for severely high versus mildly high HGI were 1.047 (95% CI: 0.648–1.689) for rehospitalization for HF, 1.398 (95% CI: 0.660–2.960) for recurrent MI, 1.083 (95% CI: 0.601–1.949) for cardiovascular death, 1.027 (95% CI: 0.622–1.697) for all‐cause death, and 1.161 (95% CI: 0.788–1.711) for MACEs. Subgroup analyses within the higher‐HGI range likewise did not reveal any significant interaction across prespecified subgroups (Figure S3).

## 4 Discussion

In this multicenter cohort of patients with AMI without reduced LVEF, we found a non‐linear association between HGI and adverse outcomes. The prognostic association was mainly observed in the lower range of HGI. Among patients with HGI below the cutoff, lower HGI was associated with higher risks of HF rehospitalization, recurrent MI, cardiovascular death, all‐cause death, and MACEs. In contrast, in patients with higher HGI, these associations were weak and were no longer significant after multivariable adjustment. Further analysis within the lower‐HGI group showed that severely low HGI was associated with higher risks of HF rehospitalization and MACEs than mildly low HGI. Overall, the prognostic value of HGI was mainly limited to the lower range.

The biological significance of HGI may help explain these findings. HGI reflects the difference between measured HbA1c and HbA1c predicted from glucose, rather than glucose alone [12]. Blood glucose and HbA1c represent different metabolic states: the former is strongly influenced by acute stress responses, whereas the latter reflects longer‐term glycemic status [13]. Mathematically, a highly negative HGI implies that the FBG is disproportionately elevated relative to the baseline HbA1c, closely reflecting the magnitude of stress hyperglycemia. Disproportionate hyperglycemia is often associated with stronger sympathetic activation, greater inflammatory stress, and more severe metabolic instability [14]. Low HGI may identify patients with a more pronounced acute injury response relative to their chronic glycemic background.

Our findings further suggest that adverse outcomes may not depend solely on absolute glucose levels, but also on the mismatch between acute hyperglycemia and chronic glycemic status. This is consistent with previous studies showing the prognostic relevance of such glycemic mismatch in patients with AMI [15, 16]. Recent evidence from NSTEMI patients undergoing PCI showed that acute‐to‐chronic glycemic mismatch, as reflected by the SHR, was independently associated with type 4a myocardial infarction and adverse outcomes [17]. These findings suggest that the discrepancy between acute glucose levels and chronic glycemic status may provide more relevant information on cardiovascular risk than either measure considered alone. SHR and the glycemic gap describe the extent to which acute glucose levels differ from chronic glycemic status [15, 18]. These indices largely reflect the acute clinical condition and are influenced by the neuroendocrine stress response during the event, the timing of admission glucose measurement, and the severity of hyperglycemia [19, 20]. In contrast, HGI quantifies the discordance between measured HbA1c and HbA1c predicted from glucose levels. Because HGI is derived from relatively stable measures, including HbA1c and FBG, it may reflect stable interindividual variation in hemoglobin glycation. This variation may be shaped by factors beyond glucose levels, including erythrocyte lifespan, red blood cell turnover, and glucose handling within erythrocytes [21]. Although low HGI, high SHR, and a positive glycemic gap may all indicate a relatively low HbA1c for a given glucose level, HGI is not equivalent to these indices of mismatch between acute and chronic glycemic status. It may capture additional prognostic information related to the individual glycation phenotype. This distinction may help explain our findings. Patients without reduced LVEF are often considered to be at lower risk; our data suggest that glycation related metabolic vulnerability may still identify a high risk subgroup. Metabolic factors may provide additional prognostic information beyond ventricular function alone.

Our results are partly consistent with previous studies, but they also differ in an important way. Earlier studies in ACS and CAD found that HGI was associated with adverse outcomes [6, 7, 8]. Some of these studies further suggested a U‐shaped or otherwise non‐linear relationship, with higher risk at both low and high HGI levels [8, 22]. We also observed a non‐linear association. In our cohort, the main prognostic signal was confined to the lower‐HGI range, whereas the higher range carried little independent prognostic information after adjustment. High and low HGI may not reflect the same pathophysiological process. High HGI may be more related to chronic abnormalities in glucose metabolism and other factors affecting HbA1c [23], whereas low HGI may be more closely linked to acute metabolic changes during stress. In CAD cohorts, especially those with a higher proportion of diabetes patients, both may be present, and a U‐shaped association may therefore be easier to observe [7, 8]. Our cohort was more selected, as patients with reduced LVEF and HF at discharge were excluded. This may have reduced the burden of advanced chronic structural and metabolic injury and made the lower‐HGI signal more apparent. Our findings do not conflict with previous reports. Instead, they suggest that the prognostic relevance of HGI may differ across patient populations [9, 10].

The subgroup findings were generally consistent with the main results. The inverse association between HGI and cardiovascular death appeared stronger in patients with LVEF ≥ 50% than in those with LVEF 41%–49%. When systolic function is relatively preserved, differences in metabolic response may become more relevant to later risk. This finding should be interpreted with caution because most other interactions were not significant and the number of events was limited in some subgroups.

The present study has several strengths. First, it was based on a multicenter cohort, which improves the reliability of the findings. Second, we focused on patients with AMI without reduced LVEF. This is an important but understudied population. Third, the combination of RCS analysis, piecewise Cox regression, and subgroup analysis allowed us to better characterize the non‐linear association between HGI and prognosis at different HGI levels. Several limitations should be noted. First, this was a retrospective observational study, and the findings should not be interpreted as causal. Potential selection bias should also be acknowledged, as HGI could only be evaluated in patients with available FBG and HbA1c data. Second, HGI was based on a single measurement at admission and may not fully reflect metabolic changes during AMI. Third, we adjusted for multiple covariates, but the effects of unmeasured or unknown confounders cannot be completely ruled out. The HGI cutoff of −0.174 was consistent across all the study endpoints but was derived from this retrospective cohort and remains unvalidated; it should therefore be considered exploratory pending confirmation in independent cohorts. Prospective studies should further examine changes in HGI during AMI and clarify its biological significance.

## 5 Conclusions

In AMI patients without reduced LVEF, HGI was non‐linearly associated with adverse outcomes, with the prognostic signal concentrated mainly in the lower HGI range. Our study was a retrospective analysis, and the findings should be viewed as exploratory. Further validation in independent cohorts is needed before HGI is applied to post AMI risk stratification.

## Author Contributions

Shangjian Luo and Huan Liu contributed to study design, statistical analysis, and drafting of the manuscript. Xuesong Wen conceived the study, supervised the work, and approved the final manuscript. All authors have read and approved the final manuscript.

## Funding

This work was supported by the Natural Science Foundation of Chongqing, China (CSTB2024NSCQ‐MSX1061).

## Ethics Statement

The study process was in accordance with the Declaration of Helsinki and was approved by the Institutional Review Board of the First Affiliated Hospital of Chongqing Medical University. Informed consent was obtained from all patients for the study.

## Consent

All authors approved the current version and agreed to the submission policies. The manuscript has not been published and is not being considered for publication elsewhere in whole or part in any language.

## Conflicts of Interest

The authors declare no conflicts of interest.

## Supporting information


**Table S1:** Comparison of baseline characteristics between the included cohort and patients excluded because of missing HbA1c, missing FBG or other key clinical data, in‐hospital death, or unavailable follow‐up.
**Table S2:** Cox regression analysis of upper‐tail HGI subgroups and clinical outcomes among patients with HGI > −0.174.
**Figure S1:** Linear regression of HbA1c on fasting blood glucose.
**Figure S2:** Kaplan–Meier curves for outcomes in patients with HGI > −0.174.
**Figure S3:** Subgroup analyses in patients with HGI > −0.174.

## Data Availability

The datasets used and/or analyzed during the current study are available from the corresponding author on reasonable request.
