# Supplementary material for: Hemoglobin Glycation Index and Prognosis in Patients With Acute Myocardial Infarction Without Reduced Left Ventricular Ejection Fraction: A Multicenter Retrospective Cohort Study
Source: J Diabetes. 2026 Jul 20;18(7):e70257. doi: 10.1111/1753-0407.70257 (PMC13386103; doi:10.1111/1753-0407.70257)
Supplement: Supplementary file 1 — Table S1: Comparison of baseline characteristics between the included cohort and patients excluded because of missing HbA1c, missing FBG or other key clinical data, in‐hospital death, or unavailable follow‐up. Table S2: Cox regression analysis of upper‐tail HGI subgroups and clinical outcomes among patients with HGI > −0.174. Figure S1: Linear regression of HbA1c on fasting blood glucose. Figure S2: Kaplan–Meier curves for outcomes in patients with HGI > −0.174. Figure S3: Subgroup analyses in patients with HGI > −0.174. [file JDB-18-e70257-s001.docx]

**Supplementary Table S1. Comparison of baseline characteristics between the included cohort and patients excluded because of missing HbA1c, missing FBG or other key clinical data, in-hospital death, or unavailable follow-up.**

| **Characteristic** | **Overall**  N = 4,071 | **Excluded** N = 2,365 | **Included** N = 1,706 | ***P*-value** |
| --- | --- | --- | --- | --- |
| **Demographic and clinical characteristics** | | | | |
| Age, (years) | 64.0 (54.0-73.0) | 64.0 (55.0-73.0) | 63.0 (54.0-72.0) | 0.106 |
| Male, n (%) | 3,112.0 (76.4%) | 1,812.0 (76.6%) | 1,300.0 (76.2%) | 0.758 |
| BMI, (kg/m^2^) | 24.1 (22.2-26.2) | 24.1 (22.0-26.2) | 24.2 (22.4-26.2) | 0.031 |
| HR, bpm | 79.0 (69.0-90.0) | 78.0 (68.0-90.0) | 79.5 (70.0-90.0) | 0.063 |
| SBP, mmHg | 129.0 (112.0-147.0) | 129.0 (112.0-147.0) | 130.0 (113.0-147.0) | 0.331 |
| DBP, mmHg | 78.0 (68.0-88.0) | 78.0 (68.0-88.0) | 78.0 (69.0-88.0) | 0.857 |
| **Comorbidities** | | | | |
| Hypertension, n (%) | 2,316.0 (56.9%) | 1,330.0 (56.2%) | 986.0 (57.8%) | 0.322 |
| T2DM, n (%) | 1,444.0 (35.5%) | 727.0 (30.7%) | 717.0 (42.0%) | <0.001 |
| CKD, n (%) | 284.0 (7.0%) | 162.0 (6.8%) | 122.0 (7.2%) | 0.710 |
| Hyperlipidemia, n (%) | 827.0 (20.3%) | 489.0 (20.7%) | 338.0 (19.8%) | 0.499 |
| CAD, n (%) | 156.0 (3.8%) | 90.0 (3.8%) | 66.0 (3.9%) | 0.917 |
| Stroke, n (%) | 331.0 (8.1%) | 196.0 (8.3%) | 135.0 (7.9%) | 0.666 |
| Smoking, n (%) | 2,519.0 (61.9%) | 1,466.0 (62.0%) | 1,053.0 (61.7%) | 0.864 |
| Cancer, n (%) | 90.0 (2.2%) | 52.0 (2.2%) | 38.0 (2.2%) | 0.951 |
| AF, n (%) | 234.0 (5.7%) | 140.0 (5.9%) | 94.0 (5.5%) | 0.579 |
| **Angiographic characteristics** | | | | |
| STEMI, n (%) | 2,669.0 (65.6%) | 1,473.0 (62.3%) | 1,196.0 (70.1%) | <0.001 |
| Anterior MI, n (%) | 1,552.0 (38.1%) | 869.0 (36.7%) | 683.0 (40.0%) | 0.033 |
| Inf/Post MI, n (%) | 1,414.0 (34.7%) | 822.0 (34.8%) | 592.0 (34.7%) | 0.971 |
| Other sites MI, n (%) | 490.0 (12.0%) | 291.0 (12.3%) | 199.0 (11.7%) | 0.536 |
| Killip class ≥ II, n (%) | 733.0 (18.0%) | 420.0 (17.8%) | 313.0 (18.3%) | 0.630 |
| CAG, n (%) | 3,755.0 (92.2%) | 2,175.0 (92.0%) | 1,580.0 (92.6%) | 0.446 |
| Thrombolytic, n (%) | 139.0 (3.4%) | 79.0 (3.3%) | 60.0 (3.5%) | 0.759 |
| PCI therapy, n (%) | 3,177.0 (78.0%) | 1,806.0 (76.4%) | 1,371.0 (80.4%) | 0.002 |
| Primary PCI, n (%) | 2,027.0 (49.8%) | 1,179.0 (49.9%) | 848.0 (49.7%) | 0.927 |
| CABG, n (%) | 11.0 (0.3%) | 5.0 (0.2%) | 6.0 (0.4%) | 0.543 |
| Reperfusion, n (%) | 3,203.0 (78.7%) | 1,820.0 (77.0%) | 1,383.0 (81.1%) | 0.002 |
| LVEF, (%) | 57.0 (52.0-61.0) | 57.0 (52.0-61.0) | 57.0 (52.0-61.0) | 0.209 |
| LVEF≥ 50%, n (%) | 3,405.0 (83.6%) | 1,971.0 (83.4%) | 1,434.0 (84.1%) | 0.577 |
| **Laboratory measurements** | | | | |
| Peak CK-MB, (µg/L) | 15.6 (3.6-53.1) | 15.6 (3.7-54.2) | 15.6 (3.6-52.0) | 0.385 |
| Peak cTnI, (ng/mL) | 2.4 (0.4-7.6) | 2.4 (0.6-7.2) | 1.5 (0.2-8.2) | <0.001 |
| BNP, (pg/mL) | 155.0 (56.0-380.5) | 125.0 (31.3-350.0) | 177.0 (79.1-397.5) | <0.001 |
| Creatinine, (umol/L) | 76.0 (64.3-92.0) | 76.0 (65.0-92.0) | 75.0 (64.0-91.0) | 0.049 |
| eGFR,(mL/min/1.73m^2^) | 93.0 (74.2-103.1) | 93.1 (74.3-103.0) | 93.0 (74.1-103.3) | 0.760 |
| LDL-C, (mmol/L) | 2.7 (2.2-3.3) | 2.7 (2.2-3.3) | 2.7 (2.2-3.3) | 0.190 |
| **Medications at discharge** | | | | |
| Anticoagulants, n (%) | 126.0 (3.1%) | 85.0 (3.6%) | 41.0 (2.4%) | 0.030 |
| Diuretic, n (%) | 727.0 (17.9%) | 424.0 (17.9%) | 303.0 (17.8%) | 0.891 |
| Antiplatelets, n (%) | 3,714.0 (91.2%) | 2,142.0 (90.6%) | 1,572.0 (92.1%) | 0.080 |
| Clopidogrel/Ticagrelor | 3,999.0 (98.2%) | 2,323.0 (98.2%) | 1,676.0 (98.2%) | 0.967 |
| DAPT, n (%) | 3,684.0 (90.5%) | 2,134.0 (90.2%) | 1,550.0 (90.9%) | 0.504 |
| Statin, n (%) | 4,009.0 (98.5%) | 2,331.0 (98.6%) | 1,678.0 (98.4%) | 0.601 |
| ACEI/ARB/ARNI | 2,800.0 (68.8%) | 1,591.0 (67.3%) | 1,209.0 (70.9%) | 0.015 |
| Beta-blocker, n (%) | 3,270.0 (80.3%) | 1,859.0 (78.6%) | 1,411.0 (82.7%) | 0.001 |
|  | | | | |

Data are presented as mean ± standard deviation (SD), median (interquartile range [IQR]), or number (percentage). Comparisons between groups were performed using the Student’s t-test or Wilcoxon rank-sum test for continuous variables, and the chi-squared test or Fisher’s exact test for categorical variables.

Abbreviations: HGI, hemoglobin glycation index; BMI, body mass index; T2DM, type 2 diabetes mellitus; CKD, chronic kidney disease; CAD, coronary artery disease; AF, atrial fibrillation; CAG, coronary angiography; PCI, percutaneous coronary intervention; CABG, coronary artery bypass grafting; LVEF, left ventricular ejection fraction; CK-MB, creatine kinase-MB; cTnI, cardiac troponin I; BNP, B-type natriuretic peptide; Glu, glucose; HbA1c, glycated hemoglobin; eGFR, estimated glomerular filtration rate; LDL-C, low-density lipoprotein cholesterol; ACEI, angiotensin-converting enzyme inhibitor; ARB, angiotensin receptor blocker; ARNI, angiotensin receptor-neprilysin inhibitor; DAPT, dual antiplatelet therapy.

**Supplementary Table S2. Cox regression analysis of upper-tail HGI subgroups and clinical outcomes among patients with HGI > -0.174.**

| **Variables** | **Mildly high HGI**  **(-0.174**<**HGI**<**0.326) (N=423)** | **Severely high HGI**  **(HGI**≥**0.326)**  **(N=422)** |  |
| --- | --- | --- | --- |
|  | HR (95%CI) | HR (95%CI) | *P-*value |
| **Rehospitalization for HF** | |  |  |
| Model 1 | Ref | 1.448 (0.966-2.170) | 0.073 |
| Model 2 | Ref | 1.427 (0.951-2.142) | 0.086 |
| Model 3 | Ref | 1.047 (0.648-1.689) | 0.853 |
| **Recurrent MI** | |  |  |
| Model 1 | Ref | 1.481 (0.800-2.743) | 0.211 |
| Model 2 | Ref | 1.562 (0.842-2.899) | 0.158 |
| Model 3 | Ref | 1.398 (0.660-2.960) | 0.382 |
| **All-cause death** | |  |  |
| Model 1 | Ref | 1.210 (0.795-1.841) | 0.374 |
| Model 2 | Ref | 1.181 (0.775-1.800) | 0.440 |
| Model 3 | Ref | 1.027 (0.622-1.697) | 0.916 |
| **Cardiovascular death** | |  |  |
| Model 1 | Ref | 1.299 (0.793-2.129) | 0.299 |
| Model 2 | Ref | 1.269 (0.773-2.085) | 0.346 |
| Model 3 | Ref | 1.083 (0.601-1.949) | 0.791 |
| **Major adverse cardiovascular events** | | |  |
| Model 1 | Ref | 1.489 (1.075-2.062) | 0.017 |
| Model 2 | Ref | 1.493 (1.076-2.071) | 0.016 |
| Model 3 | Ref | 1.161 (0.788-1.711) | 0.451 |

The mildly high HGI group was used as the reference group. Model 1 was unadjusted; Model 2 was adjusted for age, sex, and BMI; and Model 3 was further adjusted for hypertension, T2DM, hyperlipidemia, CKD, STEMI, LVEF, and primary PCI.

Abbreviations: HF, heart failure; MI, myocardial infarction; MACEs, major adverse cardiovascular events; HR, hazard ratio; CI, confidence interval; BMI, body mass index; CKD, chronic kidney disease; STEMI, ST-segment elevation myocardial infarction; LVEF, left ventricular ejection fraction; PCI, percutaneous coronary intervention.

**Supplementary Figure S1. Linear regression of HbA1c on fasting blood glucose.**

**
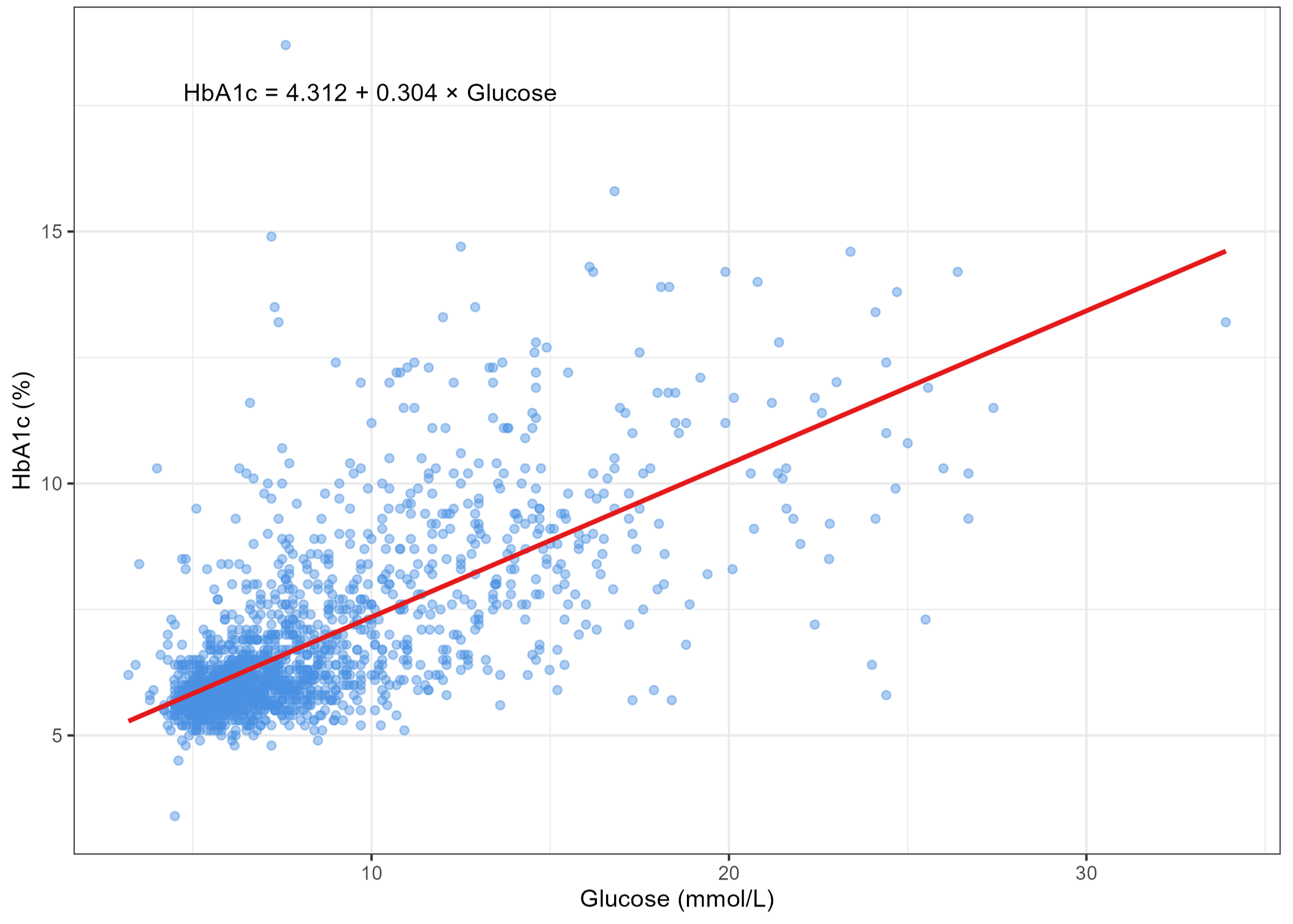
**

Scatter plot showing the linear relationship between fasting blood glucose and HbA1c. The regression equation was used to estimate predicted HbA1c for calculation of HGI. Abbreviations: HbA1c, glycated hemoglobin; HGI, hemoglobin glycation index.

**Supplementary Figure S2. Kaplan-Meier curves for outcomes in patients with HGI > -0.174.**


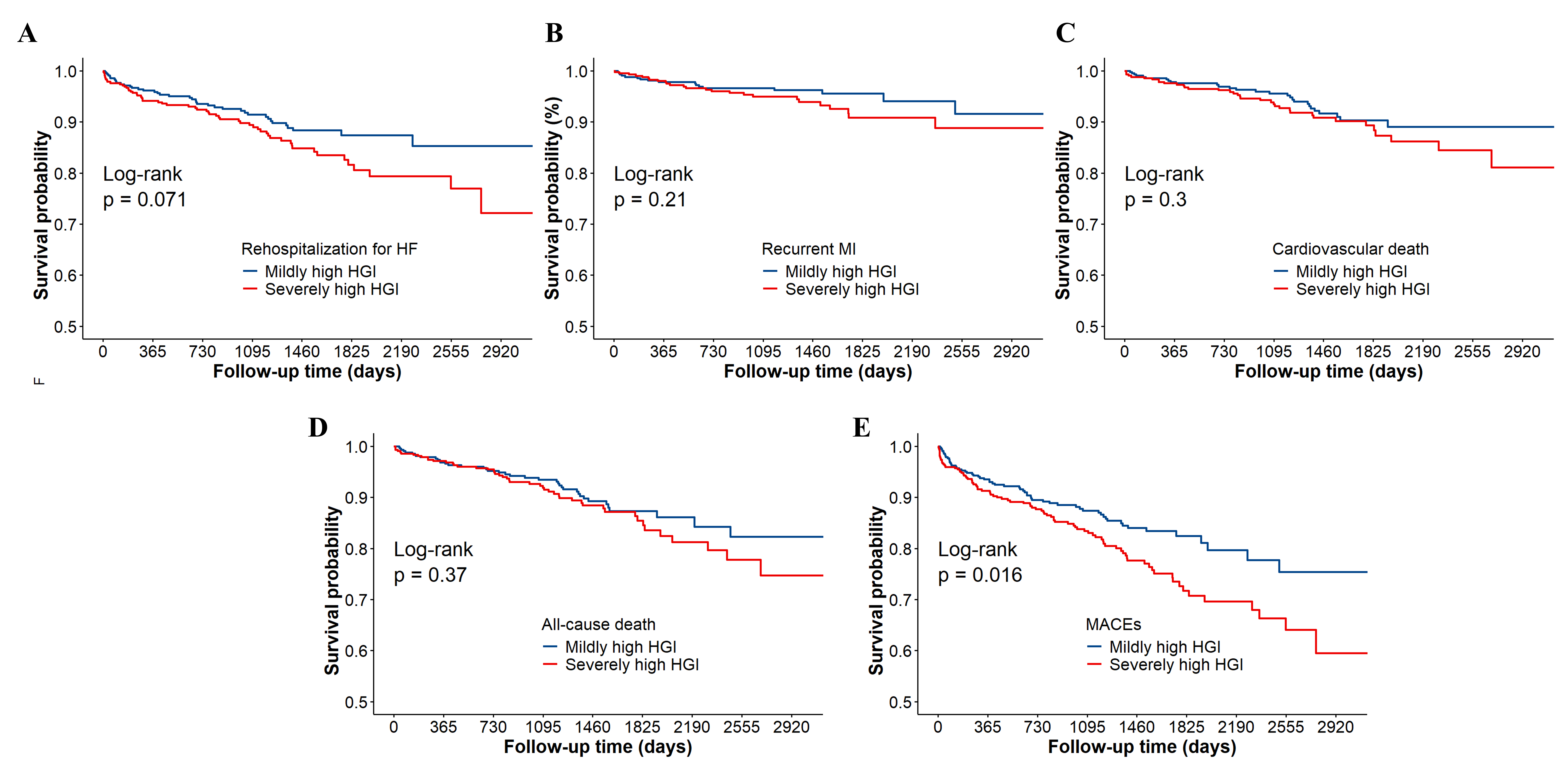


Kaplan-Meier curves for rehospitalization for heart failure (A), recurrent myocardial infarction (B), cardiovascular death (C), all-cause death (D), and major adverse cardiovascular events (E) in patients with HGI > -0.174. Patients were further divided into mildly high HGI (-0.174<HGI<0.326) and severely high HGI (HGI≥0.326) groups according to the median HGI value within this subgroup. Abbreviations: HGI, hemoglobin glycation index; HF, heart failure; MI, myocardial infarction; MACEs, major adverse cardiovascular events.

**Supplementary Figure S3.** Subgroup analyses in patients with HGI > -0.174.


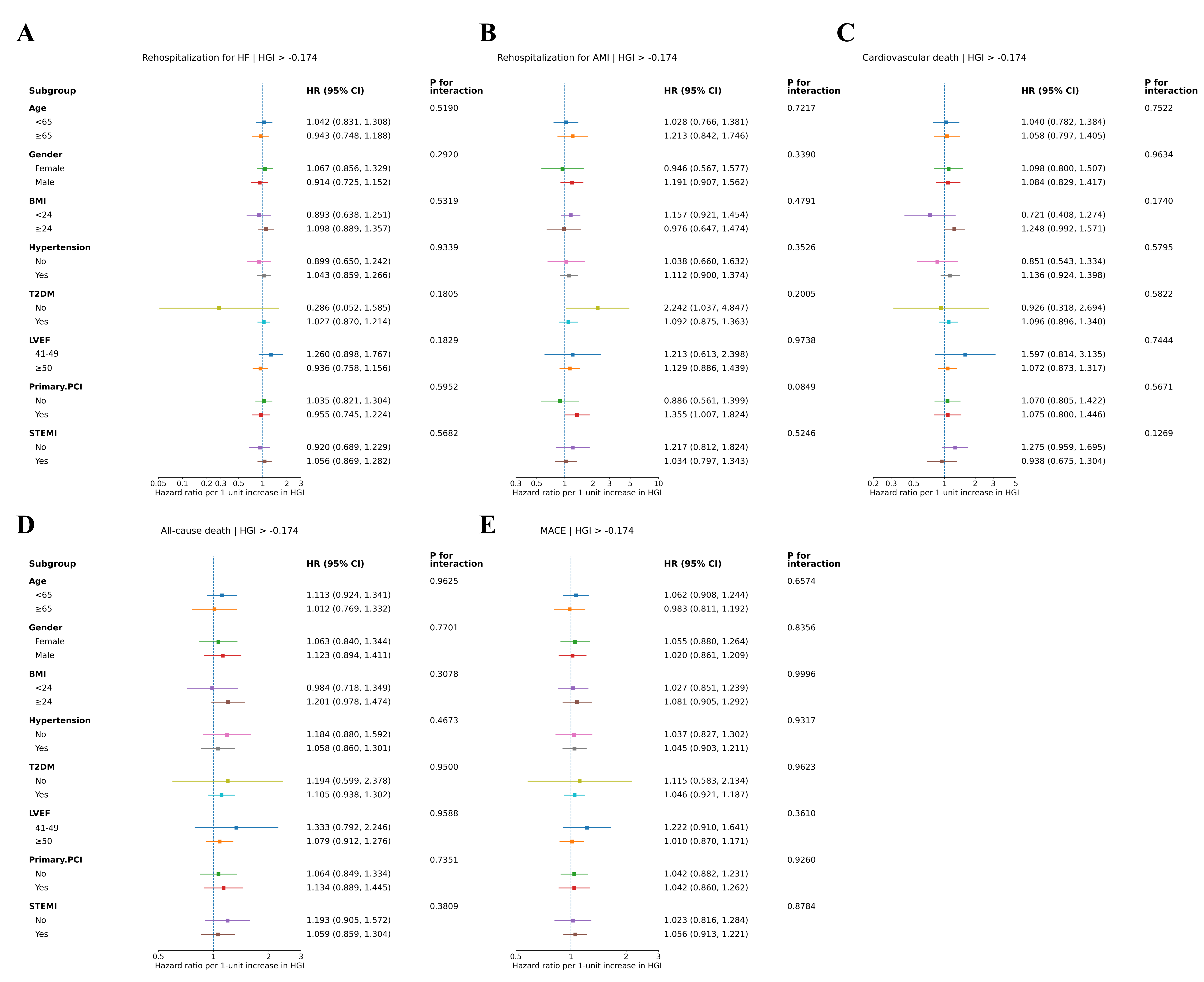


Forest plots of subgroup analyses for rehospitalization for heart failure (A), recurrent myocardial infarction (B), cardiovascular death (C), all-cause death (D), and major adverse cardiovascular events (E) among patients with HGI > -0.174. Hazard ratios with 95% confidence intervals are presented per 1-unit increase in HGI. *P* for interaction is shown for each subgroup. The model was adjusted for age, sex, BMI, hypertension, T2DM, hyperlipidemia, CKD, STEMI, LVEF, and primary PCI.

Abbreviations: HGI, hemoglobin glycation index; HR, hazard ratio; CI, confidence interval; MACEs, major adverse cardiovascular events.
